# The archaeal division protein CdvB1 assembles into polymers that are depolymerized by CdvC

**DOI:** 10.1002/1873-3468.14324

**Published:** 2022-03-09

**Authors:** Alberto Blanch Jover, Nicola De Franceschi, Daphna Fenel, Winfried Weissenhorn, Cees Dekker

**Affiliations:** ^1^ 2860 Department of Bionanoscience Kavli Institute of Nanoscience Delft Delft University of Technology The Netherlands; ^2^ CEA CNRS Institut de Biologie Structurale (IBS) Université Grenoble Alpes France

**Keywords:** Archaea, Cdv system, CdvB1, cell division, ESCRT‐III

## Abstract

The Cdv proteins constitute the cell division system of the Crenarchaea, a machinery closely related to the ESCRT system of eukaryotes. Using a combination of TEM imaging and biochemical assays, we here present an *in vitro* study of *Metallosphaera sedula* CdvB1, the Cdv protein that is believed to play a major role in the constricting ring that drives cell division in the Crenarchaea. We show that CdvB1 self‐assembles into filaments that are depolymerized by the Vps4‐homolog ATPase CdvC. Furthermore, we find that CdvB1 binds to negatively charged lipid membranes and can be detached from the membrane by the action of CdvC. Our findings provide novel insight into one of the main components of the archaeal cell division machinery.

## Graphical abstract

This study shows that the archaeal cell division protein CdvB1 can form filaments *in vitro*, and how these filaments are depolymerized by the AAA ATPase CdvC. We show that CdvB1 can bind to lipid membranes and can be detached from them by CdvC, providing new insights on the protein interactions of the archaeal Cdv system.

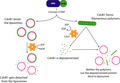

## Abbreviations

**ESCRT**, endosomal sorting complexes required for transport

**MBP**, maltose binding protein

**MIM** domain, MIT‐interacting moiety

**MIT** domain, microtubule interacting and trafficking

The Cdv system is the protein machinery responsible for cell division in the archaeal phylum of the Crenarchaeaota [1]. Many components of this cell division machinery share a high degree of homology with the eukaryotic ESCRT machinery [1, 2] that is responsible for the cell division, vesicle budding, and multiple membrane‐deforming processes in humans and yeast [3]. This has led to the suggestion that the Cdv system is an evolutionary antique and simplified precursor of the eukaryotic ESCRT machinery [4], that may share the same mechanism at its core. While the eukaryotic protein complex is well studied, the complications of imaging live thermophilic cells such as the Crenarchaeaota, that live at ~ 85 °C, has long hindered a similarly fast growth in our understanding of the Cdv system.

Up until recently, most of our knowledge of the Cdv system was limited to CdvA, CdvB (an ESCRT‐III homolog) and the AAA ATPase CdvC (Vps4 homolog), which are all found in the same operon [1]. Basically, the CdvA protein was found to form a ring at the centre of the cell together with CdvB [1]. CdvA binds to the membrane in a spiral‐like fashion [5] and acts as a membrane anchor for CdvB [6], as CdvB cannot bind to the membrane by itself [7]. Furthermore, CdvC is also found at the ring at the middle of the cell during cytokinesis [2]. In eukaryotes, several proteins of the ESCRT‐III complex interact with the AAA ATPase Vps4 through a MIM‐MIT interaction [8], and the role of the ATPase is to detach the ESCRT‐III proteins from the membrane and allow for the remodelling of the constricting filaments during the cytokinesis [9]. Prompted by the homologies between the archaeal and eukaryotic proteins, and the evidence for the presence of CdvC at the division ring during cytokinesis [2], it was thus believed that the role of CdvC in archaea is that of Vps4 in eukaryotes, namely providing energy to the system by removing ring proteins from the membrane for its remodelling during the constriction.

For a long time, the role of the CdvB paralogs (CdvB1, CdvB2 and CdvB3) was unclear. It was shown that *Sulfolobus* cells lacking CdvB were unable to grow, while cells lacking the CdvB paralogs were still viable, albeit with a lower growth rate or aberrant daughter cells [10], and therefore the CdvB1‐3 paralogs were deemed non‐essential for cell division. Recently, however, developments in high‐temperature microscopy techniques [11, 12] together with high‐resolution imaging of fixed cells [13] shed valuable light on how the CdvB paralogs participate in the cell division. It was shown that CdvB1 and B2 are recruited to the CdvB ring right before the cytokinesis, whereupon CdvB appeared to detach from the membrane as it was digested by the proteasome, while CdvB1 and CdvB2 carry out the deformation of the membrane needed for the division of the cell [11, 13]. Additionally, it was shown that *Sulfolobus* mutants without any CdvB2 undergo asymmetric cell divisions yielding differently sized daughter cells, while cells without CdvB1 occasionally failed to divide, yielding multiploid cells with two genomes [11]. All this changed the view of the basic mechanism of the Cdv system. Now the initial CdvA:CdvB ring at the centre of the cell appears to be viewed as a non‐contractile assembly ring that recruits CdvB1 and CdvB2 to the division site. In this picture, the initial ring gets digested by the proteasome and CdvB1 and CdvB2 are left to deform the membrane and perform the division of the cell.

While this renewed model for the Cdv system arises, many questions remain. The constriction of the membrane requires a continuous and controlled disassembly of the contractile ring during the membrane deformation for successful scission and division of the cell [13]. The division ring starts from a low‐curvature conformation at the beginning of the division, and needs to proceed to an invagination of the membrane all the way down to the final step of scission. In this process, molecules that initially form the ring need to be removed to allow the final scission of the membrane to occur and avoid steric hindrance at the neck of the division site. It, however, remains unclear what drives these processes of depolymerization and constriction.

Since CdvB1 contains a MIM domain at the C‐terminus, it has been hypothesized that CdvC may interact with CdvB1 through the MIT‐MIM interaction the same way that in eukaryotes Vps4 interacts with the ESCRT‐III, and CdvC may thus be responsible for the disassembly of the contractile ring [13, 14]. In *Sulfolobus islandicus*, yeast two‐hybrid screenings showed interaction between CdvC and the CdvB paralogs [15], supporting the idea of its role in the disassembly of the contractile ring. However, there has so far been no experimental evidence that shows that this interaction leads to the depolymerization of structures formed by any of the CdvB paralogs. It is also unclear how the contractile ring stays bound to the cell membrane as the CdvB paralogs lack the wH domain that allows for the interaction with CdvA [6]. Therefore, CdvB is presumed to act as a link between the membrane anchor CdvA, and CdvB1, which may start the recruitment of the contractile ring, but this raises the question of what links CdvB1 to the membrane after CdvB is gone. It has been proposed that the CdvA:CdvB ring does not fully disappear from the division site, but instead gets largely depolymerized, with a few proteins left behind which may be enough to hold the contractile ring in place [14]. However, it has also been suggested that, in contrast to CdvB, the CdvB paralogs have a protein patch homologous to the membrane‐binding domain of the human ESCRT‐III CHMP3, with a certain degree of basicity to it, which could allow them to bind the membrane directly [4].

Here, we show how the CdvB paralog CdvB1 is able to polymerize on its own. We observe that heterologously expressed and purified CdvB1 proteins spontaneously self‐assemble *in vitro* into filamentous structures. Furthermore, we show that CdvB1 filaments can get depolymerized by the action of CdvC, directly demonstrating both the presence of an interaction between these two proteins and the filament‐remodelling activity of CdvC for the first time. Finally, we investigate the lipid‐binding properties of CdvB1, and demonstrate that the ATPase activity of CdvC can detach CdvB1 from the lipid membrane.

## Materials and methods

### Plasmids

The gene for CdvB1 from *Metallosphaera sedula* (Msed_2179, UniProtID A4YIR6) was obtained from the Gen Bank database, and was reverse translated using the EMBOSS Backtranseq tool, optimized for *Escherichia coli* codon usage. To the resulting DNA sequence, a codon of a cysteine for fluorescent labelling was added at the N‐terminal of the protein, as well as Tobacco Etch Virus (TEV) and an HRV 3C proteases cutting sites (see Table S1 for full sequences). The whole gene construct was ordered as a synthetic gene already inserted in a pMAL‐c5x vector from Biomatik, using BamHI and EcoRI cutting sites. The plasmid for CdvC from *M. sedula* (Msed_1672, UniProtID A4YHC5) was kindly provided by Patricia Renesto’s lab.

### Protein purification

MBP‐CdvB1 was produced in a BL‐21 *E. coli* strain. Cells were grown at 37 °C in LB^amp^ medium to an OD of around 0.5. Expression was induced with a final concentration of 0.1 mm of IPTG for 4 h. Cells were then harvested by centrifugation at 4500 ***g*** at 4 °C for 12 min. The pellet was resuspended again in lysis buffer [50 mm Tris pH8.8, 50 mm NaCl, 50 μm TCEP, cOmplete™ Protease Inhibitor Cocktail (Roche, Basel, Switzerland)]. Cells were lysed by French press, and the lysate was then centrifuged for 30 min at 2 00 000  *g*. in a Ti45 rotor (Beckman Coulter, Pasadena, CA, USA). Supernatant was then incubated rotating with 1 mL of amylose resin (NEB, Ipswich, MA, USA) at 4 °C for 2 h. In a 4 °C room, the lysate was then poured through a gravity chromatography column, then washed twice with 1 column volume of purification buffer (50 mm Tris pH 8.8, 50 mm NaCl, 50 μm TCEP). The washed resin was incubated for 5 min with elution buffer (50 mm Tris pH 8.8, 50 mm NaCl, 50 μm TCEP, 10 mm maltose), and finally eluted the protein out using the same column. The protein was then concentrated down to a volume of 0.5 mL, and run through a Superdex™ 75 increase 10/300 GL size exclusion chromatography column mounted in an ÄKTA™ Pure system. Sample ran with purification buffer, and purity of the eluted peaks was evaluated by a 12% SDS/PAGE gel stained with Coomassie blue. The resulting MBP‐CdvB1 was snap frozen in liquid nitrogen and stored at −80 °C.

A fraction of the protein was separated after the amylose resin column elution, and dialysed into the same purification buffer but at a pH of 7.4. A maleimide‐cysteine conjugation reaction was then performed with Alexa488‐maleimide to link it to the cysteine added to CdvB1, thus obtaining fluorescently labelled CdvB1. The rest of the purification stayed the same, and excess label was removed from the protein through the gel filtration column. The resulting MBP‐CdvB1‐Alexa488 was snap frozen in liquid nitrogen and stored at −80 °C. Whenever needed for an experiment, samples were thawed at room temperature and the MBP tag was cleaved off with a 3C protease right before use.

CdvC was produced in a C41(DE3) *E. coli* strain. Cells were grown at 37 °C in LB^amp^ medium to an OD of around 0.5. Expression was induced with a final concentration of 0.1 mm of IPTG for 4 h. Cells were then harvested by centrifugation at 4500 ***g*** at 4 °C for 12 min. The pellet was resuspended in lysis buffer [50 mm Tris pH8.8, 50 mm NaCl, 1% CHAPS, 5 mm TCEP, cOmplete™ Protease Inhibitor Cocktail (Roche)]. Additional lysozyme at 1 mg·mL^−1^ was added to the resuspended cell pellet and left incubating for 1 h at 30 °C shaking. Then cells were lysed by sonication, and the lysate was centrifuged for 30 min at 45 000 r.p.m. in a Ti45 rotor (Beckman Coulter). Supernatant was loaded into a HisTrap™ HP Ni^+2^‐NTA mounted in an ÄKTA™ Pure system column for affinity purification with the His‐Tag on the CdvC. The eluted protein was then further concentrated and ran through a HiPrep Sephacryl S‐300 HR size exclusion chromatography column, using a buffer of 50 mm Tris pH8.8, 50 mm NaCl, 5 mm TCEP. Purity of the eluted peaks was evaluated by SDS/PAGE stained with Coomassie blue. The resulting CdvC was snap frozen in liquid nitrogen and stored at −80 °C.

### TEM imaging

MBP‐CdvB1 at a final concentration of 1 μm was mixed with 0.1 μm of the 3C protease in buffer containing 50 mm Tris pH 7.4, 50 mm NaCl and, to allow filaments for form for at least 1 h. The measurements of width and length of the filaments were extracted from three independent experiments. For samples with liposomes, lipids used were DOPC (1,2‐dioleoyl‐sn‐glycero‐3‐phosphocholine), DOPG (1,2‐dioleoyl‐sn‐glycero‐3‐phospho‐(1′‐rac‐glycerol)), PIP2 (1,2‐dioleoyl‐sn‐glycero‐3‐phospho‐(1′‐myo‐inositol‐4′,5′‐bisphosphate)) and Rhodamine‐PE (1,2‐dioleoyl‐sn‐glycero‐3‐phosphoethanolamine‐*N*‐(lissamine rhodamine B sulfonyl)) and they were all purchased from Avanti Polar Lipids (Alabaster, AL, USA). LUVs of 400 nm of 7 : 3 DOPC : DOPG were prepared by extruding the lipid mixture at a concentration of 5 mg·mL^−1^ through a polycarbonate filter. LUVs were then diluted down to 0.5 mg·mL^−1^, mixed with 0.1 μm of 3C protease first, and then MBP‐CdvB1 was added to a final concentration of 1 μm. It was all left to incubate at room temperature for at least 1 h. Samples were absorbed on glow‐discharged carbon‐coated 400‐mesh copper grid purchased from Quantifoil (Großlöbichau, Germany) and stained with 2% uranyl acetate. They were then imaged on a JEOL JEM‐1400plus TEM (JEOL, Akishima, Tokyo, Japan) at 120 kV of accelerating voltage with a TVIPS f416 camera (TVIPS, Gauting, Germany).

MBP‐CHMP2AΔC and CHMP3 were purified as previously described [16]. MBP‐CHMP2AΔC at 10 μm and CHMP3 at 1 μm were mixed in 50 mm Tris pH 7.4, 150 mm NaCl, 1 mm TCEP in the presence or absence of liposomes and incubated overnight. Liposomes were made with a mixture 9 : 1 of DOPC : PIP2. Samples were deposited on carbon‐coated grids and stained with 2% uranyl acetate. Images were obtained on a Tecnai 12 microscope (FEI, Hillsboro, OR, USA) at 120 kV of accelerating voltage with a Gatan Orius SC1000 camera (Gatan, Pleasanton, CA, USA).

### ATPase activity assay

The ATPase activity assay was done using the Phosphate Assay Kit – PiColorLock™ from Abcam (Cambridge, UK) and performed according to the manufacturer’s guidelines. A final concentration of CdvC of 0.1 μm in buffer 50 mm HEPES pH 7.5, 50 mm NaCl and 5 mm MgCl_2_ was tested with a concentration of 0.1 mm ATP at different temperatures (room temperature, 30 °C, 40 °C, 50 °C, 60 °C, 70 °C). The reaction was stopped at various time points (15, 30, 45 and 60 min) by submerging the samples in liquid nitrogen. Afterwards they were thawed, added the reagent of the kit and measured the absorbance at 630 nm in a 96‐well plate reader. A free phosphate standard curve was plotted to calculate the amount of phosphate released by the protease during the reaction. All experimental conditions were performed in triplicates and results were normalized to buffer with ATP under the same conditions without the ATPase.

For the comparison of CdvC ATPase activity with or without CdvB1, we mixed CdvB1 filaments at a final concentration of 1 μm with CdvC at a final concentration of 0.1 μm, in a buffer containing 50 mm HEPES pH 7.5, 50 mm NaCl, 2 mm MgCl2 and Ficoll 41.25 mg·mL^−1^. A sample was also similarly prepared without CdvB1. Right before incubation, ATP at a final concentration of 0.1 mm was added. The samples were then incubated at 50 °C for 25 min, and the reaction was quenched by putting it on ice. ATP consumption was measured using the same reaction kit and protocol as mentioned above. All experimental conditions were performed in triplicates and results were normalized to buffer with ATP under the same conditions without the ATPase.

### Sedimentation analysis of filament depolymerization

For the filament formation, 1 μm of MBP‐CdvB1 was mixed with 0.1 μm of 3C protease in buffer 50 mm HEPES pH 7.4, 50 mm NaCl, 5 mm MgCl_2_, Ficoll 41.25 mg·mL^−1^. Higher concentrations of Ficoll slightly diminished the activity of CdvC (Fig. S4) so a final concentration of 41.25 mg·mL^−1^ was chosen, as this provided enough thermal stability but did not greatly reduce the CdvC ATPase activity. Incubated overnight at 4 °C to guarantee full formation of the CdvB1 filaments. The next day, mix in 0.6 μm of CdvC and incubate for 30 min. For the depolymerization of the filaments, ATP was added to a final concentration of 1 mm and then incubated at 50 °C for 2 min in a thermocycler. The same reactions with either ATPγS or ADP instead of ATP were performed in parallel as control experiments. After incubation, the sample was transferred to an ultracentrifuge tube and spun down in a Ti 42.2 rotor at 140 000 ***g*** for 30 min at 4 °C. After centrifugation, the supernatant was collected, the pellet was resuspended in the same volume, and they were analysed by SDS/PAGE stained with Coomassie.

### Liposome flotation assay for membrane binding

Protocol adapted from Ref. [17]. Lipids were mixed to final ratios (mol : mol) of 99.9 DOPC : 0.1 Rhodamine‐PE or 69.9 DOPC : 30 DOPC : 0.1 Rhodamine‐PE and evaporated in a glass vial to a final amount of 500 μg. They were later resuspended in 100 μL of buffer containing 50 mm HEPES pH 7.5, 50 mm NaCl and 300 mm sucrose. The lipid film was hydrated for 1 h and thoroughly vortexed to form small lipid vesicles. In an ultracentrifuge tube, 300 μg of the lipid vesicles were mixed with 3C protease and MBP‐CdvB1‐Alexa488, to a final concentration of 1.5 μm. Lipids and protein were left to incubate for 45 min, and then buffer with sucrose was mixed to obtain a bottom layer of 80 μL of 30% sucrose solution. Carefully, on top of it, a layer of the same volume of buffer with 25% sucrose was deposited, and another layer with 0% sucrose buffer on top of all. Then it was centrifuged at 200 000 ***g*** at 21 °C for 30 min in a Ti 42.2 rotor. Finally, the lipid and middle layers (fractions 1 and 2) were pipetted out, the remaining buffer was removed and the pellet at the bottom of the tube was resuspended in fresh buffer (fraction 3). All different fractions were analysed by SDS/PAGE. The acrylamide gel was imaged with a GE Amersham™ Typhoon gel imager (GE, Chicago, IL, USA) to observe the fluorescence of protein and lipids.

### Detachment of CdvB1 from the membrane by CdvC

Binding of CdvB1 to the membrane was performed as described for the “Liposome Flotation Assay” but in a PCR tube instead of the ultracentrifuge tube. CdvC was added to the CdvB1 at a final concentration of 0.8 μm, and the result was added to either ATP at a final concentration of 1 mm, or simply to the storage buffer (50 mm HEPES pH 7.5, 50 mm NaCl, and 20 mm MgCl_2_). Samples were incubated for 10 min at 50 °C in a thermocycler. After incubation, samples were moved to ultracentrifuge tubes and the sucrose gradient and subsequent centrifugation were performed as described in the liposome flotation assay for membrane binding.

### Assessment of the binding of pre‐formed CdvB1 polymers

Polymers of CdvB1 were formed by cleavage of the MBP in absence of any lipids. MBP‐CdvB1 at 14 μm was mixed with 1 μm of 3C protease, and left incubating at RT. After 1 h, the filaments were spun down at 70 000 ***g*** for 20 min to separate the filaments from the remaining monomers. Then, the protein was mixed with the same amount of liposomes and CdvC as for the previous CdvB1 depolymerization experiments, and the depolymerization reaction was carried out by adding ATP at 50 °C for 10 min. After that, the sample was left at room temperature to allow any binding of the proteins for 45 min, and then the sucrose gradient and subsequent centrifugation were performed as previously described. Samples were then analysed by SDS page the same way as for the previous experiment of membrane detachment.

## Results

### CdvB1 self‐assembles into polymeric filaments

**Fig. 1:**
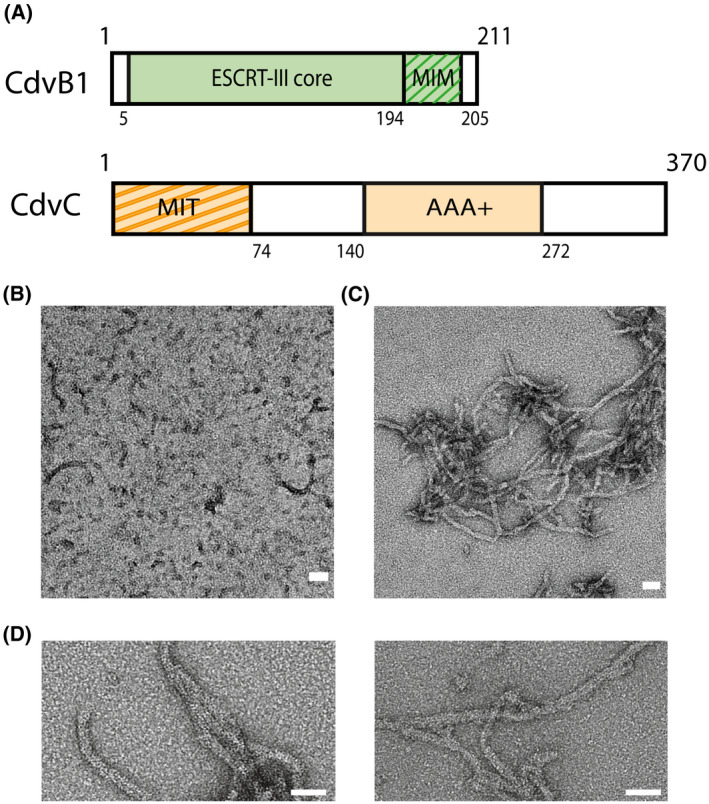
CdvB1 can polymerize into filamentous polymers (A) Schematic representation of CdvB1 and CdvC. (B) Negative staining EM image of MBP‐CdvB1 monomers and some short polymers. (C) Negative staining EM image of the filamentous polymers formed by CdvB1 upon cleavage of the fused MBP. (D) Closeup image of the CdvB1 polymers. All scale bars 50 nm.

CdvB1 and CdvC proteins from the archaeon *M. sedula* (Fig. 1A) were heterologously expressed in *E. coli* and purified (see Materials and methods). To improve the handling and solubility of the protein, CdvB1 was fused to an MBP tag at the N‐terminus of the protein, with an HRV 3C protease cleavage site in between the two. After the purification, the MBP was initially left on the protein, which largely impeded the polymerization of the protein, as can be seen by negative‐staining TEM images (Fig. 1B).

It has been previously reported that CdvB, like many other ESCRT‐III proteins, switches between an active and an inactive state when it comes to polymerization [6, 16]. More specifically, CdvB contains a self‐inhibiting domain that prevents it from polymerizing, while it spontaneously forms filaments when this domain is removed. We observed that this is not the case for CdvB1. Upon cleavage of the MBP by the 3C protease, CdvB1 spontaneously self‐assembled into elongated filamentous polymers (Fig. 1C), without the need of any other protein or the removal of any domain. The filaments have a defined width of about 14 ± 2 nm and a variety of different lengths, presenting an average length of 320 ± 140 nm (Fig. 1D). The filaments tended to stick to each other and thus form filamentous aggregates, making it difficult to properly measure the length when exceeding the 500 nm.

### CdvB1 polymers are disassembled by CdvC

**Fig. 2:**
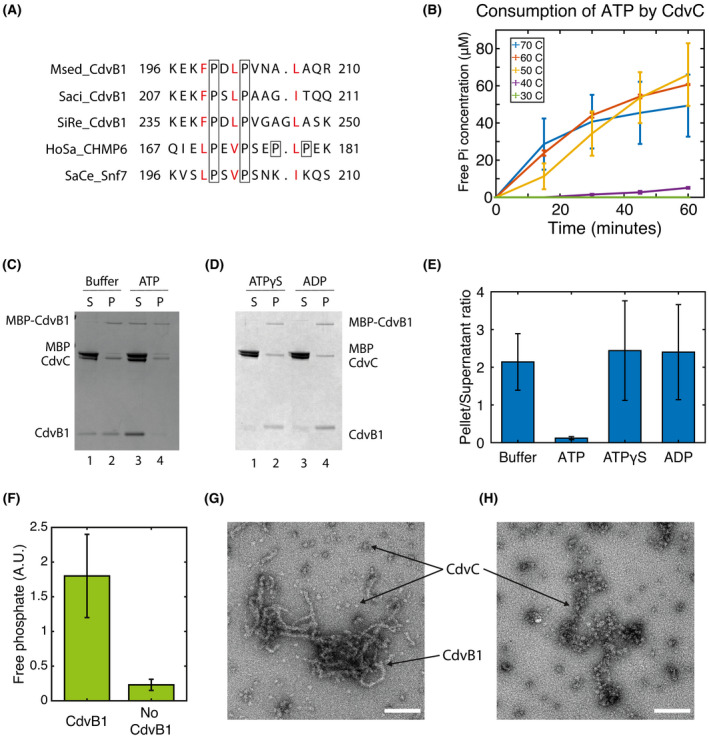
Filaments of CdvB1 are depolymerized by CdvC. (A) Sequence alignment of regions of CdvB1 from Metallosphaera sedula (Msed2179), Sulfolobus islandicus (SiRe1200), and Sulfolobus acidocaldarius (Saci_0451), with the MIM2 domain of human ESCRT‐III protein CHMP6 and yeast Saccharomyces cerevisiae Snf7. Conserved hydrophobic residues (red) and the prolines (boxed) are highlighted. (B) ATP consumption by CdvC at different temperatures. (C) SDS page analysis of a sedimentation assay where the depolymerization of CdvB1 filaments is assessed. (D) SDS page analysis of a sedimentation assay where ATPgS and ADP were added to the sample instead of ATP. No depolymerization of CdvB1 is observed. (E) Quantitative analysis of the Pellet‐to‐Supernatant ratios shown in the sedimentation assays (n=3). (F) Comparison of the ATP consumed by CdvC in 25 minutes at 50°C in the presence or absence of CdvB1 G. Filaments of CdvB1 with CdvC after incubation at 50°C but without ATP. H. EM image showing the disappearance of the CdvB1 filaments when mixed with CdvC, ATP and incubating at 50°C, leaving only aggregates of CdvC behind. All scale bars 50 nm.

Next, we studied whether these polymers of CdvB1 can get depolymerized by the action of CdvC. It has been hypothesized that CdvB1 can interact with CdvC, as some components of the ESCRT‐III complex possess a MIM domain (MIT‐interacting moiety) at the C‐terminus of their sequence, which interacts with the MIT domain of the CdvC/Vps4 ATPase. The sequences of these two interacting domains are highly conserved among species, and the CdvB1 of various different crenarchaea exhibit, at the C‐terminus of their sequence, a high degree of homology with the MIM2 domains of human’s CHMP6 or the yeast’s Snf7 (Fig. 2A). The MIM2 domain of *M. sedula* is practically identical to that of *S. islandicus* (Fig. 2A), organism in which CdvB1 and CdvC have been shown to interact through yeast 2 hybrid assays [15]. Furthermore, the MIM2 consensus sequence shows that prolines and hydrophobic amino acids are highly conserved at specific locations [18], and thus CdvB1 has a high degree of sequence similarity to the human and yeast proteins.

The high temperature where the Crenarchaea live in their natural habitat posed an experimental challenge for testing the CdvB1‐CdvC interaction. It had been previously reported that, *in vitro*, CdvC is enzymatically active at temperatures above 60 °C [6]. In our *in vitro* experiments, the CdvB1 polymers were broken down when incubated for prolonged times at temperatures above the 40 °C, so we decided to verify whether a compromise between the ATPase activity of CdvC and the thermal stability of the CdvB1 polymers could be found. As shown in Fig. 2B, CdvC did, as expected, not show any activity at temperatures up to 40 °C. However, the protein did show a significant activity already at 50 °C. At that temperature, together with the addition of Ficoll crowder, the CdvB1 polymers remained stable (Fig. S1), and hence, we chose this as our working temperature in the experiments.

To investigate if this interaction causes CdvC to disassemble CdvB1 filaments, we first formed CdvB1 polymers by cleaving off the MBP tag and allow them to polymerize. CdvC was then added to the samples, together with either ATP, ADP, non‐hydrolysable ATP (ATPγS), or buffer without nucleotides (Buffer). These samples were incubated for 2 min at 50 °C, to allow for the ATPase activity of CdvC, samples were centrifuged down at high speed, and pellet and supernatant were separately run on a gel. As can be seen from Fig. 2C, polymerized CdvB1 was forming pellets at the bottom during the centrifugation, while monomeric CdvB1 remained in the supernatant (Fig. 2C, lines 1 and 2). However, when adding ATP and therefore allowing CdvC to act, the pelleted fraction virtually disappeared, and most of the protein was found to be in the monomeric state (Fig. 2C, lines 3 and 4). The CdvB1 pellet was also still present when, instead of ATP, non‐hydrolysable nucleotides were provided to the reaction (Fig. 2D). The filament‐to‐monomer ratio of ~ 2 was the same for the samples where no hydrolysable nucleotides were added (ADP or ATPγS), whereas that ratio was drastically lowered to a value of 0.1 when CdvC could consume ATP (Fig. 2E). Several independent experiments were performed to corroborate this (Fig. S1). The depolymerization of CdvB1 did not occur when there was no CdvC present in the reaction (Fig. S2), indicating that the heating step did not disrupt the CdvB1 polymers, and it was indeed caused by CdvC.

To see if the depolymerization of the CdvB1 filaments is indeed the result of a specific interaction between CdvC and the CdvB1 filaments, we checked the level of ATPase activity of CdvC in the presence or absence of CdvB1 filaments. We observed a very clear increase in the ATPase activity in the presence of CdvB1, showing a consumption of ATP that was eight times larger than for CdvC alone (Fig. 2F). This suggests that the MIM domain of CdvB1 acts as a substrate for CdvC, which can bind it and perform its activity of depolymerizing the CdvB1 polymers, which consumes additional ATP.

We visualized the depolymerized filaments through negative staining EM. CdvB1 filaments were formed, CdvC was added and incubated at 50 °C, as described above, and samples were imaged. When no hydrolysable nucleotide was added, CdvB1 filaments were observed in the sample, together with CdvC oligomers around them (Fig. 2G). However, in samples where ATP had been added, the filaments of CdvB1 had vanished and only CdvC aggregates were observed (Fig. 2H). Together with the evidences observed in the previous sedimentation assays, we thus conclude that the action of CdvC was responsible for the depolymerization of the CdvB1 filaments.

### CdvB1 binds negatively charged lipid membranes and can be detached by CdvC

**Fig. 3:**
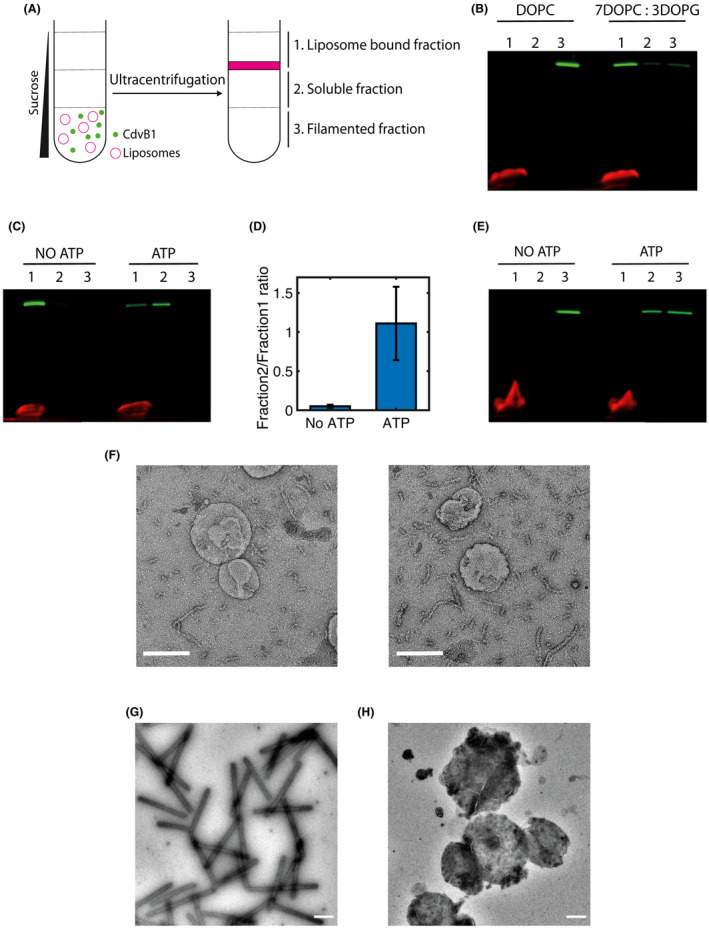
CdvB1 monomers bind to negatively charged lipid membranes but CdvB1 polymers do not. (A) Schematic of the liposome flotation assay. (B) SDS page gel of the liposome flotation assay showing CdvB1‐Alexa488 in the liposome fraction only when the assay is performed in the presence of negatively charged lipids (30% DOPG). (C) Liposome flotation showing that CdvB1 protein bound to liposomes is detached from the lipid membrane by CdvC. (D) Quantitative analysis of the protein detachment from the membrane, showing the soluble‐to‐lipid‐bound ratio (n=3). (E) Liposome flotation assay showing that CdvB1 protein filaments do not bind to liposomes. The monomers resulting from their depolymerization do not bind either. (F) Negative‐staining EM image showing that the CdvB1 protein filaments do not interact with the membrane of the liposomes. Scale bars 200 nm. (G) Negative‐staining EM image of human ESCRT III proteins CHMP2A and CHMP3 co‐polymerizing into tube‐like structures. Scale bar 500 nm. (H) Negative‐staining EM image showing that CHMP3 does not form tube‐like polymers when incubated with liposomes. Scale bar 500 nm.

Since the Cdv proteins are involved in remodelling membranes, it is of interest to study their membrane‐binding properties. First, we studied if CdvB1 was able to directly bind lipid membranes. We used a liposome flotation assay, which, through a gradient of different concentrations of sucrose, allows distinguishing between the membrane‐bound protein (that colocalizes with the liposomes), CdvB1 monomers (that stay in solution) and CdvB1 filaments (that precipitate to the bottom) (Fig. 3A). We mixed MBP‐CdvB1‐Alexa488 with liposomes in a solution that contained the 3C protease. After incubation, we deposited the sample at the bottom of a 3‐step sucrose gradient that we centrifuged at high speed. This resulted in three different fractions (Fig. 3A): a top one where the liposomes were found (1), a middle one with monomeric protein (2) and a bottom one containing the filamentous CdvB1 (3). We tested liposomes made of DOPC + 0.1% Rhodamine‐PE and a mixture of 70% DOPC + 30% DOPG + 0.1% Rhodamine‐PE (percentages denote molar fractions) to examine the effect of the negative charges of the DOPG against the neutrality of DOPC. All the fractions of the gradient were analysed by SDS/PAGE where we imaged the fluorescence of both the lipids (red) and the CdvB1‐Alexa488 (green). What we observed was that, as hypothesized, CdvB1 never bound to liposomes that were exclusively made of DOPC. However, when DOPG was present in the mixture, the CdvB1 protein showed clear binding to the liposomes (Fig. 3B). This shows that it is not only CdvA that can bind lipid membranes, but that other components of the Cdv system can do so as well, similar to the way that different proteins of the ESCRT machinery in eukaryotes present different membrane‐binding capabilities [19].

In view of this, and the depolymerization of CdvB1 by CdvC that was described above, we tested whether CdvC was able to detach CdvB1 from the lipid membrane. For this we used the same liposome flotation assay where we bound the protein to negatively charged liposomes, subsequently added CdvC to the sample, incubated with ATP and Mg^2+^ for 10 min at 50 °C, and then deposited the sample in the 3‐step sucrose gradient. As can be seen in Fig. 3C, we observed that the CdvB1 protein remained bound to the membrane in samples with no ATP. By contrast, samples containing ATP showed a big portion of the protein that disassembled from the liposomes to go into the soluble fraction (Fig. 3C). On average, about half of the protein that was bound to the membrane depolymerized in solution in our experimental conditions (Fig. 3D). When no CdvC was added to the reaction, this detachment was not observed (Fig. S3).

This lipid‐binding behaviour was additionally tested for conditions where first CdvB1 filaments were allowed to form in the absence of liposomes, gently centrifuged to separate them from leftover monomers, then mixed with the liposomes, left to incubate for 1 h at room temperature, and finally deposited and centrifuged in the 3‐step sucrose gradient. Interestingly, we observed that, for these conditions, all of the CdvB1 was found in the bottom layer of the sucrose gradient, corresponding to the filamentous state, while no CdvB1 was found in the liposome fraction (Fig. 3E). This shows that CdvB1 filaments, once formed, did not bind the membrane.

We then tried to see if depolymerization of the CdvB1 filaments with CdvC would allow the newly solubilized CdvB1 to bind the lipids. For this, we formed CdvB1 filaments in the absence of lipids, mixed them with 7 : 3 DOPC : DOPG liposomes, CdvC, and ATP with Mg^2+^, and incubated for 10 min at 50 °C. After the depolymerization reaction, we left the proteins with the liposomes rest at room temperature to interact for 1 h, and then performed the 3‐step sucrose gradient. We observed that neither the filaments nor the depolymerized CdvB1 showed binding to the liposomes (Fig. 3E). This may suggest that CdvC is actually unfolding the monomers of CdvB1 in the process of depolymerizing the filaments, and that the resulting depolymerized proteins lack a functional folded structure. Negative‐staining TEM images of MBP‐CdvB1 incubated with vesicles that contained the 3C protease in solution showed filaments of CdvB1 that were lying next to the vesicles (Fig. 3F). Hardly ever were these filaments found on top of the vesicles or attached to them, consistent with the results from the liposome flotation assay.

Given the similarities of the archaeal Cdv proteins and the eukaryotic ESCRT, we wanted to see if this duality between polymerization and membrane binding was present in both systems. Interestingly, a similar behaviour was indeed observed for the CHMP2A and CHMP3, which are human homologs of CdvB1 [4]. These proteins from the human ESCRT‐III machinery are well known for their *in vitro* co‐polymerization into large helical structures that can be disassembled by the ATPase Vps4 [20, 21]. These tube‐like structures easily form when mixing MBP‐CHMP2AΔC and CHMP3 at a molar ratio of 10 : 1 (Fig. 3G). However, when trying to polymerize these tubes in the presence of liposomes (9 : 1 DOPC : PIP2), we found no tubular polymerization, as seen in Fig. 3H. Instead, we observed that the protein remained bound to the surface of the liposomes, but it would never polymerize into helical tubes and bind to liposomes at the same time.

## Discussion

In this paper, we clarified a number of characteristics of the important but so far understudied Cdv protein CdvB1. We found that, *in vitro*, CdvB1 self‐assembles into filaments without the need of removing any inhibiting domain, like in many of its ESCRT‐III homologs. Fusion to an MBP impeded activation and filament formation, which is a convenient way of controlling the polymerization when needed, facilitating its study in *in vitro* assays. We also showed how CdvB1 polymers are disassembled by CdvC, showing for the first time a direct proof of depolymerization of an archaeal ESCRT‐III polymer by the action of the AAA ATPase CdvC. As ATPγS and ADP did not promote filament depolymerization, it appears that hydrolysis of ATP is needed to perform the task. Interestingly, hydrolysis of ATP by CdvC greatly increased in presence of CdvB1, suggesting that there is a specific MIM‐MIT interaction between the two proteins, which stimulates the activity of CdvC, much in the same way that the MIM domain proteins stimulate Vps4 in eukaryotes [22]. These findings strengthen the idea that the Cdv system can be considered as a relatively simpler version of the homologous eukaryotic ESCRT machinery, thus reinforcing evidence for a mechanistic common ground between the archaeal and eukaryotic cell division systems.

Our data show that CdvB1 has the ability to directly bind membranes with negatively charged lipids without the need of any anchoring proteins (such as CdvA), which contrasts previous findings for CdvB. Furthermore, CdvC was found to remove the protein from the membrane. This may explain how CdvB1 can stay attached to the membrane during the cell division after the removal of CdvB. This is consistent with the view that the initial CdvA:CdvB ring merely serves as a scaffold for the recruitment of CdvB1 to the division site, whereupon the contractile ring can stay bound to the membrane by its own interaction with the lipids after the initial ring is removed. It was reported that the initial ring gets digested by the proteasome before the constriction of the cell [13], leaving only a contractile ring of CdvB1 and CdvB2 to perform the division. Our data suggest that CdvC is responsible for inducing the depolymerization of the contractile ring that is needed for the division of the cell to occur [13, 14].

**Fig. 4:**
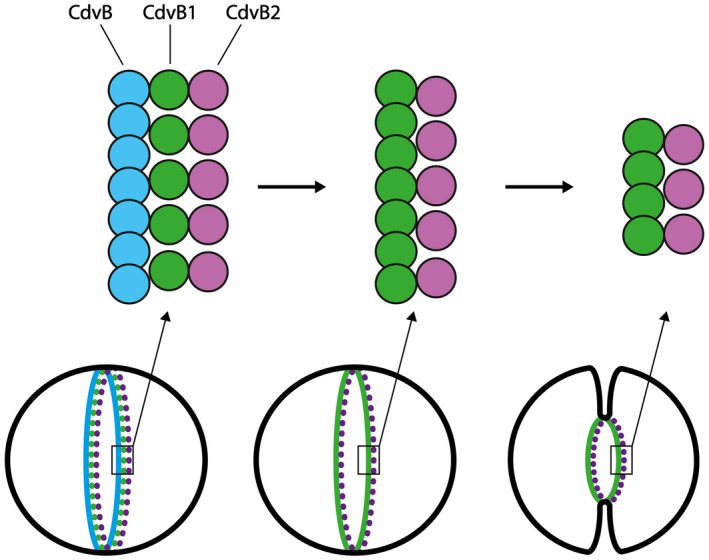
Proposed filament structure in crenarchaeal cell division. First CdvB forms a non‐contractile ring, whereupon it recruits monomeric CdvB1 and CdvB2. Upon removal of CdvB, CdvB1 can polymerize while remaining in the right place thanks to the action of CdvB2, and jointly they constrict the membrane.

We also reported an interesting duality for CdvB1, as the polymerization of the protein and its membrane‐binding capabilities were found to be mutually exclusive phenomena. After cleavage of the MBP tag, i.e. at the point where the protein is in its fully native conformation, one of two things can happen: either a CdvB1 protein finds the lipid membrane and binds to it, or it binds other CdvB1 proteins to polymerize into filaments that subsequently are not able to bind the lipid membrane. Interestingly, we showed a very similar behaviour for the human ESCRT‐III CHMP2A and CHMP3, that co‐polymerize into large helical structures in the absence of lipids, but fail to do so when surrounded by liposomes. These data might be taken to suggest that upon polymerization, the membrane‐binding patch of the proteins does not face the outside anymore, and thus loses its lipid‐binding ability. However, this seems counter‐intuitive regarding the function of the ESCRT‐III proteins that form filamentous polymers that can be reshaped to deform the membrane. We suggest that instead, *in vivo*, CdvB1 is recruited in a monomeric state to the lipid membrane at the division site by CdvB, whereupon it may form filaments (Fig. 4). This would allow CdvB1 to bind to the membrane by its membrane‐binding domain, where it may also recruit CdvB2 to the division site. There is a very high degree of conservation of positively charged amino acids between CdvB1 and CdvB2, which leads us to think that CdvB2 will likely bind to negatively charged lipid membranes as well. Therefore, we speculate that upon removal of CdvB from the membrane, with CdvB1 and CdvB2 left to constrict the membrane, CdvB1 may polymerize into filaments that will be stabilized and kept bound to the membrane by CdvB2. The action of CdvC may then remodel the CdvB1 filaments whereupon the membrane shrinks, while CdvB2 keeps the ring in place. This idea is in line with previous findings [11] where mutants lacking CdvB1 were less able to perform the fission of the membrane, indicating its role in membrane deformation, whereas mutants without CdvB2 would lead to asymmetric divisions, suggesting its guiding role.

An evolutionary correlation of these phenomena seems to be implied in the clear *in vitro* polymerization versus membrane binding that we observed in both the human and archaeal ESCRT‐III proteins. Previous studies showed the importance of conformational changes of ESCRT proteins bound to a lipid membrane, and how the different protein states, either bound or unbound, facilitated different interactions [23]. A similar scenario likely applies to the Cdv proteins where, depending on the interaction with the membrane, different polymerization states may occur. Future high‐resolution studies of the proteins *in vivo* will help to better understand how the proteins arrange themselves at the division site to facilitate faithful cell division in the Crenarchaeaota.

## Author contributions

A.B.J. Performed all experiments with the Cdv proteins and analysed the data. N.D.F. was involved in the conception of the experiments with the Cdv proteins, performed the experiments with the eukaryotic proteins D.F. assisted with the TEM imaging of the experiments with eukaryotic proteins. W.W. conceived the work with eukaryotic proteins. C.D. initially conceived the study and guided the experimental progress. The manuscript was written by A.B.J, N.D.F and C.D. with input from the other authors.

## Supporting information


**Fig. S1.** Examples of other independent experiments of depolymerization of CdvB1 by CdvC that were analyzed in Fig.2CD.

**Fig. S2.** Pelleting assay performed to samples containing only CdvB1 incubated at 50 °C, where no depolymerization of CdvB1 filaments was visible.

**Fig. S3.** Membrane depolymerization control without any CdvC, where no depolymerization is visible in any case after the incubation at 50 °C with and without ATP.

**Fig. S4.** Consumption of ATP by CdvC after 25 minutes at 50°C with different Ficoll concentrations.

**Table S1.** Sequence of MBP‐CdvB1 plasmid

Supplementary Material

## Data Availability

The data that support the findings of this study are available on request from the corresponding author.
