# Supplementary figures and images for: The archaeal division protein CdvB1 assembles into polymers that are depolymerized by CdvC
Source: FEBS Lett. 2022 Mar 9;596(7):958–69. doi: 10.1002/1873-3468.14324 (PMC9542132; doi:10.1002/1873-3468.14324)

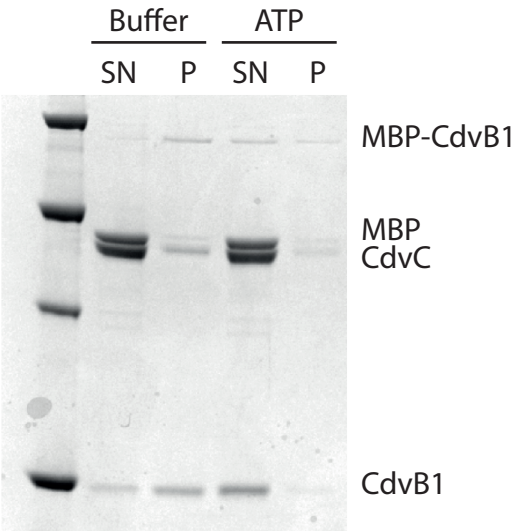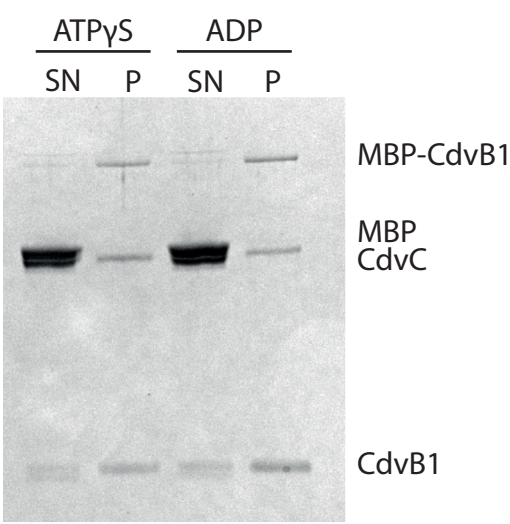

Supplement: Supplementary file 1 — Fig. S1. Examples of other independent experiments of depolymerization of CdvB1 by CdvC that were analyzed in Fig.2CD. [file FEB2-596-958-s002.pdf]

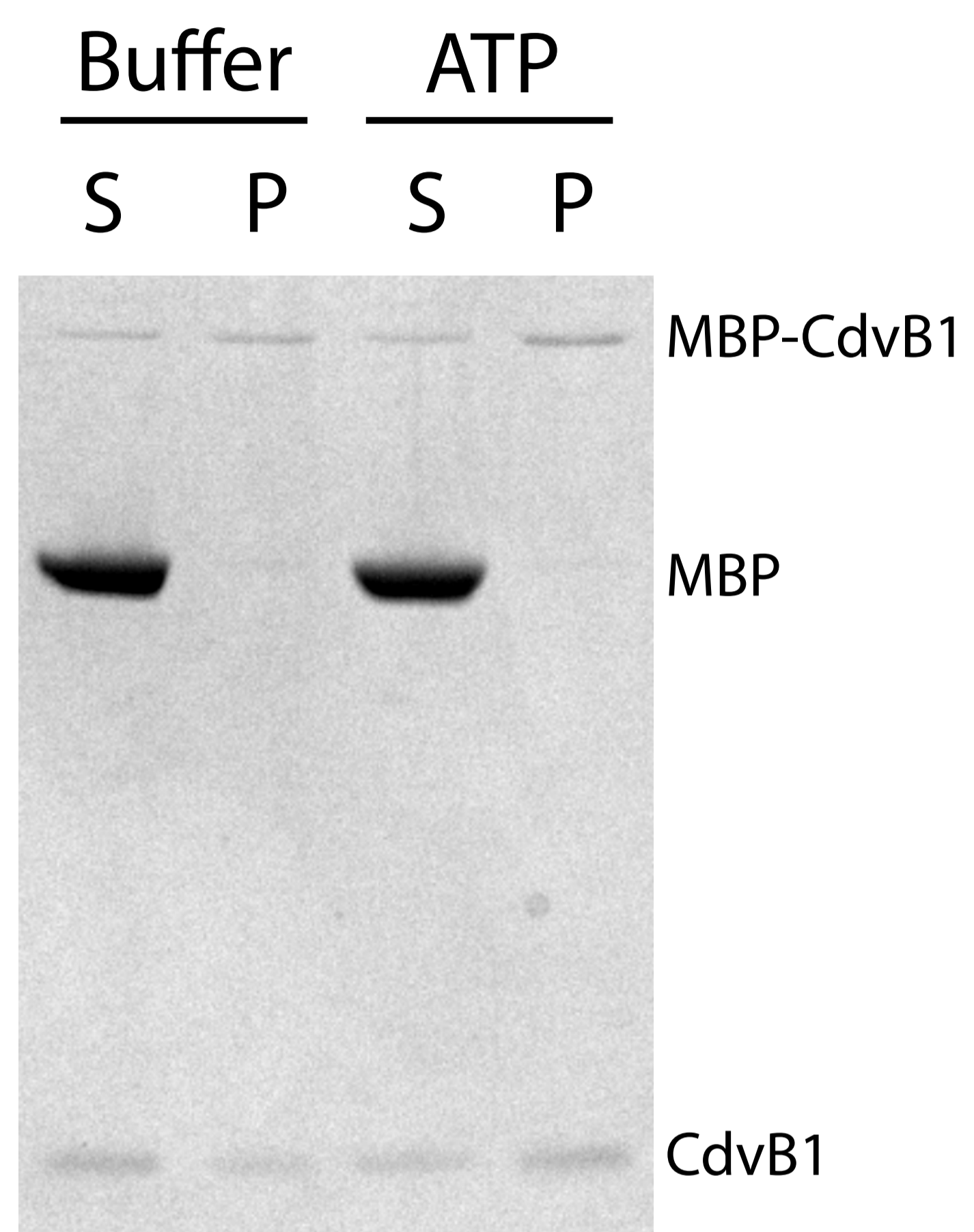

Supplement: Supplementary file 2 — Fig. S2. Pelleting assay performed to samples containing only CdvB1 incubated at 50 °C, where no depolymerization of CdvB1 filaments was visible. [file FEB2-596-958-s004.pdf]

| NO ATP |   |   | ATP |   |   |
|--------|---|---|-----|---|---|
| 1      | 2 | 3 | 1   | 2 | 3 |

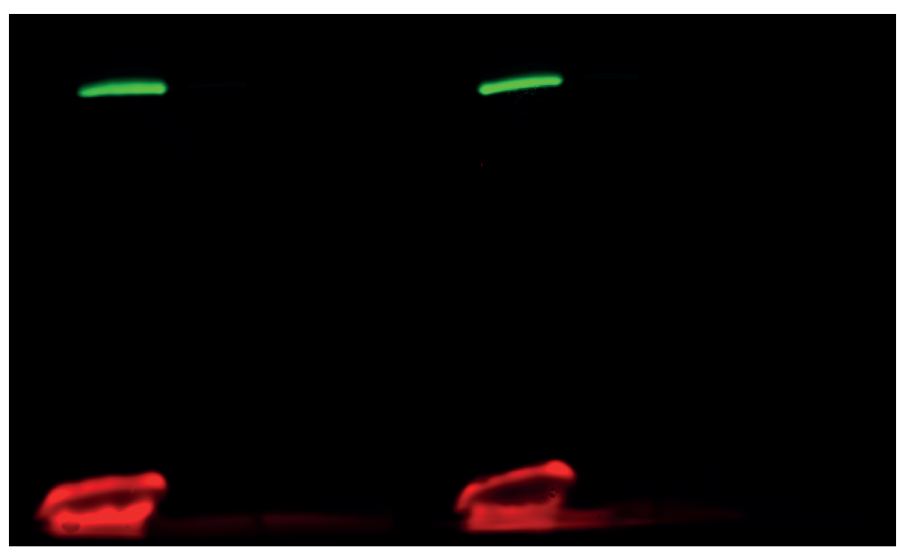

Supplement: Supplementary file 3 — Fig. S3. Membrane depolymerization control without any CdvC, where no depolymerization is visible in any case after the incubation at 50 °C with and without ATP. [file FEB2-596-958-s006.pdf]

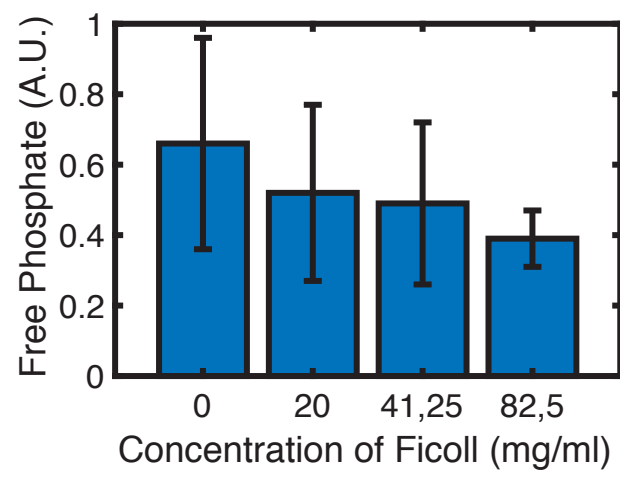

Supplement: Supplementary file 4 — Fig. S4. Consumption of ATP by CdvC after 25 minutes at 50°C with different Ficoll concentrations. [file FEB2-596-958-s001.pdf]
