# Supplementary material for: The archaeal division protein CdvB1 assembles into polymers that are depolymerized by CdvC
Source: FEBS Lett. 2022 Mar 9;596(7):958–69. doi: 10.1002/1873-3468.14324 (PMC9542132; doi:10.1002/1873-3468.14324)
Supplement: Supplementary file 6 — Supplementary Material [file FEB2-596-958-s003.docx]

**Supplementary Figure 1**

Examples of other independent experiments of depolymerization of CdvB1 by CdvC that were analyzed in Fig.2CD.

**Supplementary Figure 2**

Pelleting assay performed to samples containing only CdvB1 incubated at 50 °C, where no depolymerization of CdvB1 filaments was visible. The addition of ATP to the CdvB1 samples increased the aggregating trend of filaments, which can be seen in the more intense pellet when ATP was added with respect to the CdvB1 only sample.

**Supplementary Figure 3**

Membrane depolymerization control without any CdvC, where no depolymerization is visible in any case after the incubation at 50 °C with and without ATP. Fraction 1 shows liposome bound proteins, fraction 2 shows proteins in solution and 3 filamented proteins.

**Supplementary Figure 4**

Consumption of ATP by CdvC after 25 minutes at 50°C with different Ficoll concentrations. A slightly lower activity of the protein is observed at higher Ficoll concentrations.
